# On the Treatment of Dysentery

**Published:** 1852-07

**Authors:** C. W. Davis

**Affiliations:** Carlisle, Ill.


ART. III.—*On the Treatment of Dysentery.* By C. W. DAVIS, M.D., of Carlisle, Ill.

IN examining the pages of the *N. W. Med. and Surg. Journal,* I discover several articles on the subject of dysentery, the treatment of which seems to be considerably varied, all marked by success. I observe, however, that in the articles referred to *calomel* seems to stand first as a remedial agent. The dysentery of last summer (in this region) commenced with bilious diarrhœa, attended with considerable tenesmus, followed by discharges most generally of a muco-bloody character, very frequent, fœtid and painful, ushered in frequently by nausea and vomiting. The ejections were generally bile combined with matter resembling the white of an egg. The febrile state made its appearance early, sometimes of an inflammatory, but generally of a congestive, type, indicated by the inefficiency of purges, the capillary congestion of the extremities, and quickness and weakness of the pulse.

In the first few cases under my care, I used emetics, calomel, opium, fomentations, blisters, injections, &c. Under the administration of calomel and opium the evacuations were principally bilious; but as soon as the medicine ceased operating, the muco-bloody discharges made their appearance in redoubled violence, hence I concluded that the dejections were consequent upon an acrid and vitiated condition of the bile, flowing in inordinate quantities, and coming in contact with the mucous membrane of the large intestines causing softening of the membrane, and hence the muco-bloody discharges. I then concluded that the administration of calomel was (according to the old adage) adding fuel to fire, and ceased giving it altogether. With the view of keeping up a gentle laxative condition of the intestinal canal, without exciting the liver to an increased action, I selected such articles as I thought best adapted for the purpose, like the following:

℞—Sulph. magnesia ℥j
Super-tartrate of potassa ℥ss
Sulph. morph. grs. ij.

Mixed in 5 or 6 oz. of gum water. A tea spoonful to be taken every hour.

If I found the patient laboring under the narcotic effects of the morphine I would add more magnesia and less morphia, and vice versa. I used also stimulating applications to the extremities, fomentations, injections, &c., as the case might require.

This treatment was uniformly successful.

